# Risk of Pneumoconiosis in Workers Exposed to Crystalline Silica from Lava Rock Dust from Mount Etna

**DOI:** 10.3390/jcm14113781

**Published:** 2025-05-28

**Authors:** Francesca Vella, Veronica Filetti, Serena Matera, Salvatore Spinello, Denis Vinnikov, Giuseppe Muscato, Venerando Rapisarda, Davide Matera, Lucia Rapisarda, Ermanno Vitale

**Affiliations:** 1 Department of Clinical and Experimental Medicine, University of Catania, 95123 Catania, Italy; francescav.89@libero.it (F.V.); serena.matera@yahoo.it (S.M.); vrapisarda@unict.it (V.R.); lucirapisarda@yahoo.it (L.R.); 2 Federazione Italiana Medici di Medicina Generale-F.I.M.M.G. (Italian Federation of General Practitioners), 00153 Rome, Italy; salvospinello@gmail.com; 3 Faculty of Medicine and Health Care, Environmental Health Science Lab, Al Farabi Kazakh National University, Almaty 050040, Kazakhstan; denisvinnikov@mail.ru; 4 Occupational Health Risks Lab, Peoples’ Friendship University of Russia (RUDN University), Moscow 117198, Russia; 5 Department of Clinical and Experimental Medicine, Regional Referral Center for Rare Lung Diseases, University Hospital Policlinico “G. Rodolico-San Marco”, University of Catania, 95123 Catania, Italy; gpp.muscato@gmail.com; 6 Division of Orthopedics, Mediterranean Oncology Institute, 95029 Viagrande, Italy; damat70@gmail.com; 7 Department of Medicine and Surgery, University of Enna “Kore”, 94100 Enna, Italy; ermanno.vitale@unikore.it

**Keywords:** pneumoconiosis, silica, dust, Etna

## Abstract

**Background:** Pneumoconiosis is a group of lung diseases characterized by the deposition and accumulation of dust or mineral fibers in the lung interstitium, primarily caused by occupational exposure. **Methods:** The aim of this study was to identify cases of pneumoconiosis induced by occupational exposure in patients living on the island of Sicily (South Italy), through the retrospective analysis of pneumoconiosis cases registered by the Reference Center for the Prevention, Diagnosis and Treatment of Interstitial Lung Diseases and Rare Lung Diseases (RCLD) of University of Catania, during the decade 2012–2022. Furthermore, the results of a screening conducted in the same 10-year period (2012–2022) on cohorts of workers potentially exposed to RCS generated by Etna’s volcanic dust are reported. **Results:** From the consultation of the RCLD database, there does not appear to be any correlation between pulmonary fibrosis and exposure to dust of basaltic origin. **Conclusions:** These data seem to be confirmed by the data of the health monitoring conducted over 10 years on 200 workers involved in different processes of working with lava stone.

## 1. Introduction

Pneumoconiosis is a group of lung diseases characterized by the deposition and accumulation of dust or mineral fibers in the lung interstitium, primarily caused by occupational exposure [1]. These diseases result from the inhalation of airborne particles that penetrate and deposit in the bronchioles and alveoli [1]. The pathophysiological genesis is a chronic pulmonary inflammation that can promote pulmonary fibrosis, and then lead to pneumoconiosis [2]. Pneumoconiosis can manifest in various forms, with different types associated with specific occupational exposures [3]. One notable and extensively studied pneumoconiosis is silicosis, which is caused by the inhalation of crystalline silica dust particles containing respirable-sized silicon dioxide (SiO_2_) particles [4,5].

Crystalline silica is one of the most abundant minerals in the earth’s crust and a basic component of sand, soil, and granite [6]. Environmental exposure to respirable crystalline silica (RCS) can occur not only in various natural phenomena such as volcanic explosion and sandstorms but also in agricultural areas and a series of industries, such as metal mining, pottery process, quarrying, construction, and clay manufacturing [7]. RCS particles are respirable when they are less than 5 microns in diameter and when inhaled are capable of reaching the distal airways and alveoli and can scar the lungs [8].

Silicosis has been recognized as an occupational disease with a long history, particularly affecting individuals involved in mining and the processing of natural stone [6,9]. Occupational exposure to RCS occurs during various activities, such as mining, cutting, grinding, and working with materials that contain silica, including stone, sand, minerals, and rocks [6,9]. Chronic exposure to respirable RCS particles can lead to progressive lung tissue damage, resulting in the formation of fibrotic pattern and respiratory function impairment [5].

Several studies have highlighted that the risk of developing pneumoconiosis is not uniform across all occupations but strongly depends on the specific job role and the intensity and duration of exposure to RCS. For instance, stone workers and quarrymen often exhibit higher rates of silicosis compared to general construction workers, due to continuous and direct interaction with silica-containing materials [6,10]. Furthermore, artificial stone workers are known to be at extremely high risk due to the elevated quartz content and poor ventilation in processing environments [5]. Differences in exposure patterns and work processes significantly affect disease incidence and severity [3]. These findings underline the importance of analyzing occupational subgroups separately when assessing exposure risk.

The aim of this study was to identify cases of pneumoconiosis induced by occupational exposure in patients living on the island of Sicily (South Italy), through the retrospective analysis of pneumoconiosis cases registered by the Reference Center for the Prevention, Diagnosis and Treatment of Interstitial Lung Diseases and Rare Lung Diseases (RCLD) of University of Catania, during the decade: 2012–2022. Furthermore, the results of a screening conducted in the same 10-year period (2012–2022) on cohorts of workers potentially exposed to RCS generated by Etna’s volcanic dust are reported.

## 2. Subjects and Methods

### 2.1. Sample from RCLD

A retrospective observational study was conducted by consulting the database of RCLD for patients who were registered/visited during the decade: 2012–2022.

The RCLD established by the Regional Government collects all cases of rare lung diseases and pulmonary fibrosis that are detected by 13 public hospitals and 31 private hospitals spread across the territory (province of Catania: 1,078,766 inhabitants). The RCLD collects confirmed diagnoses of interstitial lung diseases and pneumoconiosis from hospitals and pulmonology outpatient clinics across Eastern Sicily. All cases are clinically validated by board-certified pulmonologists based on patient history, imaging (X-ray and/or CT), and pulmonary function tests, in accordance with international diagnostic criteria. The registry does not include self-reported cases.

The following keywords were used in the database search: “marble”, “stonemason”, “silicosis”, “volcanic”, “pneumoconiosis”, “dust”, “fibers”, “stone”, “worker”, “occupational exposure”, “sand”, “azole”, and “lava stone”. All cases of occupational-related interstitial lung disease were selected. Inclusion criteria were as follows: occupational exposure to dust and diagnosis of lung fibrosis. The exclusion criterion was not having had occupational exposure to dust. Subjects (cases) in which information on occupational exposure was unclear or missing were excluded.

This study was approved (n. 18580 on 30 April 2020) by the Ethics Review Board of the University Hospital Ethics Committee and written informed consent was obtained from each participant.

For each subject, all the general information (age, height, weight, voluntary habits, etc.) and medical information were collected, including remote and recent medical history, with particular reference to respiratory system diseases. A specific structured questionnaire was administered to each subject to investigate the risk factors present in the work environment [11]. In particular, information was collected on the workplace, type of agents/substances to which they had been exposed, use of mechanical tools, use of personal protection equipment (PPE), etc. Cigarette smoking history was collected as current smokers, ex-smokers (those who had not smoked for >1 year), and non-smokers (those who had never smoked). The pack-year parameter was calculated to establish the tobacco load that the patient has used during his smoking life. Respiratory symptoms such as cough, sputum, wheezing, dyspnea, etc., were collected according to the American Thoracic Society [12]. The respiratory symptoms had to be present for at least 3 months during the preceding 2 years [13].

In the case of deceased or unavailable patients, a family member was contacted to administer the questionnaires.

The body mass index (BMI) was calculated using the weight/height ratio squared (kg/m^2^). For each selected case, the medical record and instrumental and haematological examination were analyzed and taken into consideration. Data from respiratory function tests (spirometry test), diagnostic imaging (X-ray and CT) of the lung, and the results of alveolar–pulmonary diffusion of carbon monoxide (DLco) were acquired. In particular, the following respiratory parameters were detected: forced vital capacity (FVC), forced expiratory volume in the first second (FEV1), FEV1/FVC ratio, peak expiratory flow (PEF), maximal expiratory flow (MEF) rate at 25–75% of the vital capacity (VC), and total lung capacity (TLC), measured and expressed as a proportion of European Coal and Steel Community (CECA) reference values adjusted for individual characteristics (age, weight, and height) recorded at the time of testing [14].

Diagnostic imaging tests allowed us to classify pneumoconiosis according to the International Labour Organization (ILO): category I, II, and III based on the size, profusion, and distribution interval of the opacity radiologically detected during chest examination [15]. Profusion score and the predominant shape and size of the opacities were recorded; median results of the readings were reported.

### 2.2. Workers Occupational Exposed to Volcanic Dust

Data from health surveillance of three cohorts of workers exposed to volcanic dust operating in different companies in the Etna area are reported.

In Italy, the health surveillance of these workers is mandatory by law (legislative decree no. 81/2008). This activity consists of primary prevention aimed at preventing the onset of occupational diseases and secondary prevention to diagnose any pathologies early; this occurs through medical visits and instrumental tests, aimed at investigating the health status of workers, with particular attention to the functional evaluation of organs and systems defined as “critical” with respect to exposure to risks [16]. The same legislation also requires risk assessment, which also includes the execution of environmental sampling.

The three cohorts of workers exposed to volcanic dust were (1) construction workers (CW); (2) lava stone cutters/processors (LSW); and (3) lava stone quarries workers (QW).

The inclusion criteria were as follows: being workers exposed to volcanic dust, in the same sector for at least 5 years.

The exclusion criteria were as follows: being workers operating in the Biancavilla (town located on the slopes of the Etna volcano) area; being workers operating with lavas coming from the Biancavilla area; being residents in Biancavilla. This is because the Biancavilla area and the lava rocks coming from it have been contaminated by asbestos-form fibers called fluoro-edenite [17].

For each subject, all the general information (age, height, weight, voluntary habits, etc.) and medical information were collected, including remote and recent medical history, with particular reference to respiratory system diseases. The BMI (kg/m^2^) was calculated and the following tests were performed for each subject: medical examination, routine blood tests, spirometry (FVC, FEV1, FEV1/FVC ratio, PEF, MEF rate at 25–75%, VC, and TLC), and X-ray examination. Level II tests were as follows: DLco and chest CT with and without contrast medium.

All level II tests were performed in the case of suspected organ dysfunction after the medical examination/instrumental examination.

During the medical examination, a structured questionnaire (Supplementary Materials) was administered to assess professional exposure, in particular, the use of PPE, processing with dust abatement equipment, type of stone materials processed, and type of processing, including the use of tools, etc. [11].

Participation in the study took place on a voluntary basis during the mandatory health surveillance activities, according to Italian Law Decree (DL) 81/08, from January 2012 to December 2022. All workers invited to take part in the project were informed about the objectives and procedures of this study. It was not necessary to receive confirmation from the Ethical Committee as the activity is governed by the Law Decree (DL) 81/08.

### 2.3. Exposure Measurements RCS

Exposure assessment was possible for all cohorts through mandatory environmental monitoring by law in Italy (Law Decree no. 81/08). From each company participating in the study, respirable silica exposure measurement data were collected. The sampling method has already been used in a previous study [11]. It is briefly reported below that exposures were determined from personal full-shift exposure measurements. To represent a full workday, measurement times ranging from 240 to 600 min were corrected to an 8 h time-weighted average (TWA). For respirable dust and respirable silica, the detection limits were 0.10 mg per sample and 0.005 mg per sample, respectively. This resulted in concentration detection limits for an 8 h TWA sample of approximately 0.10 mg/m^3^ for respirable dust and 0.005 mg/m^3^ for respirable silica. Personal sampling measurements were performed in the workers’ breathing zone. For the sampling of respirable silica dust, an SKC aluminium cyclone (SKC 225-01-01, Eighty-Four PA, USA), with a 0.8 μm in pore size acetate filter (Millipore) and an air pump (SKC AirCheck 2000, Eighty Four PA, USA, MSA Escort, Pittsburgh, USA, or GSA SG4000, Gut Vellbrüggen, Neuss, Germany) operating at a constant airflow rate of 2.5 L/min was used. Analyses of the filters were made gravimetrically for respirable dust and by X-ray diffraction for respirable silica with the diffraction angles 2θ = 20, 26, and 50 degrees. The analyses were performed by the Department of Occupational and Environmental Medicine at Catania University, Italy.

Furthermore, through the RCS monitoring values of each company, the worker’s specific occupation, the first year of employment for all occupations involving RCS, and the time since work initiation (years), it was possible to calculate two parameters: the cumulative RCS exposure (CRCSE) and CRCSE silicosis rate—cumulative silicosis rate (CSR), as described by Raanan et al. [5]. The term CRCSE is defined as the average RCS in mg/m^3^ multiplied by the exposure period (years) and measured in mg/m/y. The CSR is the silicosis rate in a study/CRCSE and measured in % silicosis rate/mg/m^3^/y.

### 2.4. Statistical Analysis

The collected data were included in a bespoke database. Descriptive statistics were carried out to describe the sample of the study. Data were tested for normality with the Kolmogorov–Smirnov test. The association between the different variables was analyzed with the chi-square test (X^2^) or Fisher’s exact test, Student’s *t*-test, and ANOVA. The variables were considered statistically significant when *p* < 0.05. The statistical study was carried out with SPSS software (IBM SPSS Statistics for Windows, Version 23.0. Armonk, NY, USA: IBM Corp).

## 3. Results

### 3.1. RCLD Group

**Figure 1:**
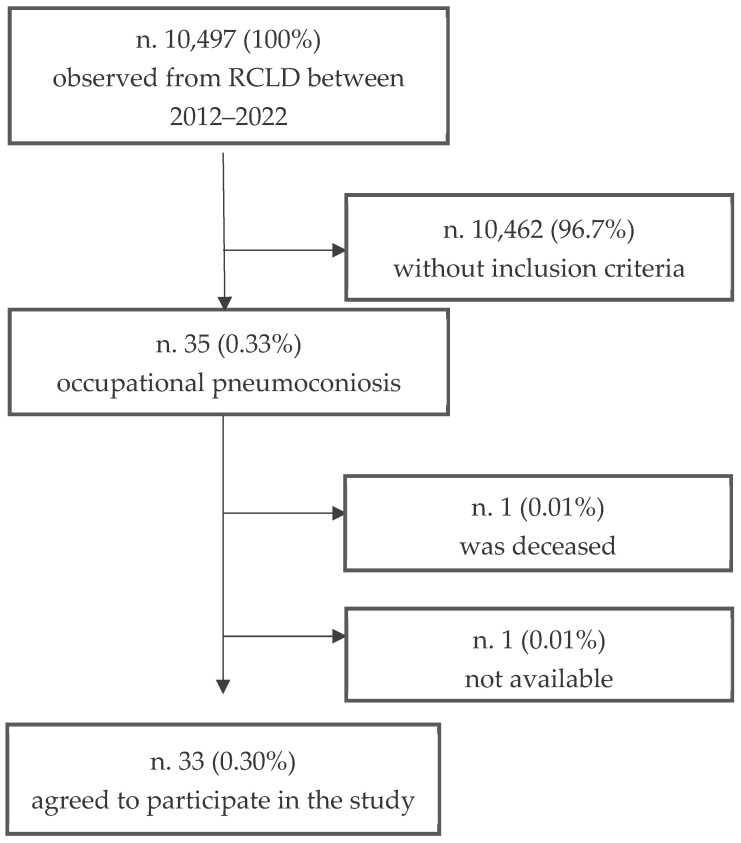
Flowchart for recruiting patients registered on RCLD.

On a total of 10,497 (100%) patients managed from RCLD in the period 2012–2022, n = 35 (0.33%) of patients had a clear occupational dust exposure and had developed pneumoconiosis. Of these 35 cases examined, only 94% (n = 33) agreed to participate in this study, undergoing the administered structured questionnaire; in the remaining 2 (6%) cases: 1 (2%) was deceased, his family not found, and 1 (2%) was not available (Figure 1).

The mean age was 66.9 ± 5.7 years and length of service was 35.4 ± 7.3 years; the mean age at diagnosis was 52.8 ± 6.1 years. The sample consisted of male workers only. At the time of diagnosis, 31 (94%) workers were smokers. The 33 (100%) workers recruited were all retired and had worked in the following sectors: 6 (18%) quarry workers (QW); 9 (27%) stone workers (SW); and 18 (55%) construction workers (CW). All the workers interviewed had been equipped with PPE for the airways, but only in the last few years (2–5 years) of work. In 27 (82%) cases, exposure to RCS deriving from machining dust was clearly detectable; in the case of 14 (42%) CW, exposure to artificial stone dust for more than 5 years was detectable. In the case of the 9 stone cutters (SW), all had a history of constant exposure to marble dust and only sporadic processing of lava stone was detectable in only 2 (6%) cases; in 4 (12%) QW, exposure to sandstone (2) and marble (2). Lava stone processing was present in a few cases: 3 (9%) CW and only 2 (6%) QW. Table 1 reports the main characteristics of patients registered in the RCLD grouped by work category.

Pulmonary function parameters (FEV_1_, FEV_1_%, FVC, FVC%, and PEF) were found to be significantly lower in all the silicosis patients and the percentage reduction in each respiratory function parameter was directly proportional to the degree of profusion category of the ILO (*p* < 0.005). The comorbidities present were cardiovascular diseases (34% vs. 33% vs. 35%; n.s.); diabetes and endocrine diseases (11% vs. 13% vs. 10%; n.s.); osteoarticular diseases (52% vs. 47% vs. 53%; n.s.); and diseases of the urogenital tract (4% vs. 6% vs. 7%; n.s.).

### 3.2. Workers Occupational Exposed to Volcanic Dust

A total of 200 (100%) occupational workers exposed to volcanic dust were studied in the period 2012–2022: n = 110 (55%) were CW; n = 56 (28%) were LSW; and n = 34 (17%) were QW.

All the workers (100%) were male; the mean age was 41.4 ± 6.2 years and length of service was 15.9 ± 5.8 years. A total of 162 (81%) were smokers, and the mean BMI was 26.9 ± 2.1. All the interviewed workers had been equipped with PPE for the airways, but only in the last 10 years (range 1–10) of work. In particular, all the exposed workers were provided with a helmet, a filter mask for personal respiratory protection, protective clothing, and gloves. In the case of the CW, most participants (95%) also reported exposure to wood dust (30%), chemicals (30%), glass and rock wool (20%), and asbestos (5%). Table 2 reports the main characteristics of the sample grouped by work categories: CW, LSW, and QW.

The samples of workers exposed to volcanic dust grouped into work categories: CW, LSW, and QW, were homogeneous in terms of anthropometric characteristics, smoking habits, and work history. No significant alterations in laboratory tests were detected in all the study participants. Elevated blood pressure values were found in 17 (8.5%) workers, which required drug therapy in 13 (6.5%) cases. In addition, 3 (1.5) workers were undergoing therapy for hypothyroidism and 2 (1%) for non-insulin-dependent diabetes mellitus. Osteoarticular pathologies were detected in 21 (10.5%) cases: (2) spondylo-discopathies of the cervical spine; (10) spondylo-discopathies of the lumbo-sacral spine; (4) rotator cuff syndrome; and (5) previous meniscal lesions. The results of the respiratory function tests and instrumental investigations are summarized in Table 3.

The instrumental investigations conducted on the three categories of workers show an average respiratory function always within the normal range. First-level diagnostic imaging investigations (chest X-ray in two views) were always within the normal range. Only in 15 (7.5%) cases was further investigation with a second level chest CT examination requested. In particular, in 2 (1%) cases, outcomes of previous pleurisy were detected; in 10 (5%) cases, mild thickening of the bronchoalveolar network in the hilar area in heavy smokers; and in 3 (1.5%) cases, outcomes of previous COVID-19 pneumonia with typical ground-glass images. In no case was it necessary to proceed with DLco.

### 3.3. Volcanic Dust Occupational Exposure

From each company participating in the study, dust and respirable silica exposure measurement data were collected. The data obtained were always below the limit values established by Italian and European legislation of 0.1 mg/m^3^ (Annex XLIII D.L 81/2008) for RCS and 3 mg/m^3^ (TLV-TWA ACGIH) for respirable dust.

In particular, the mean values measured in the three categories of workers were similar (not statistically significant) for RCS: CW = 0.0033 ± 0.0019, LSW = 0.0035 ± 0.0021, and QW = 0.0034 ± 0.0018, while there was a statistically significant difference in the mean concentrations of respirable dust: CW = 0.195 ± 0.056, LSW = 0.265 ± 0.087, and QW = 0.198 ± 0.064. In particular, stonecutting workers (LSW) had a higher concentration of respirable dust without presenting greater risks than the concentration of RCS.

The mean data on RCS and CRSE also indicate low levels of exposure risk: CW = 0.11 ± 0.09 (RCS) and 1.21 ± 0.37 (CRCSE); CW = 0.16 ± 0.19 (RCS) and 1.34 ± 0.52 (CRCSE); and CW = 0.14 ± 0.16 (RCS) and 1.30 ± 0.45 (CRCSE), with no statistically significant difference between the three subcategories.

## 4. Discussion

Volcanoes and sands are major sources of silica. It has been reported that approximately 9% of the world’s population lives within 100 km of active volcanoes [7,18] and more than 30% of the population has been exposed to sandstorms [9]. The adverse health effects of silica exposure are an increasing public health concern [19,20].

In industrial areas, more than 30 million workers have been exposed to silica worldwide [9]. Long-term exposure to silica dust has been confirmed to be associated with elevated mortality from silicosis, chronic obstructive pulmonary disease (COPD), and other airway diseases [21].

Currently, out of a total of 3335 (100%) quarries in Italy, 247 (7%) are active in Sicily (www.ISTAT.it), 148 (60%) in the Catania Mining District. Of these, 74 (30%) are located in the Etna area, where due to geographical connotation there is intense processing of lava stone from the volcano (www.regione.sicilia.it).

In many Italian regions, the use of natural stone for the construction of houses and for urban building works is frequent; in particular, this phenomenon is notably present in Sicily, an island in the south of Italy [22,23]. The geological composition of this island is different in the western part compared to the eastern part. In fact, from clayey soils we move to soils rich in white stone (limestone, sandstone, etc.) until we reach the volcanic soils of Etna, but also of smaller volcanoes, such as the Aeolian Islands [24]. In particular, in the metropolitan area of Catania, which has over 1 million inhabitants, and a significant part of eastern Sicily, the utilization of natural rocks, particularly lava stone sourced from quarries around Mount Etna, is widespread in the construction of houses and infrastructure [25].

From the analysis of data on a large population (n = 10,497) of subjects affected by pulmonary fibrosis, a negligible percentage of cases (n = 35, 0.33%) was observed for which there had been professional exposure to dust.

Analyzing the aforementioned cases, it was observed that the majority of these (n = 18, 55%) were CW, compared to SW (n = 9, 27%) and QW (n = 6, 18%).

Of the CW, 14 (42%) had been exposed to artificial stone dust for more than 5 years. In the case of the nine stone cutters (SW), all had a history of constant exposure to marble dust and only sporadic processing of lava stone was detectable in only two (6%) cases; in four (12%) QW, exposure to sandstone (2) and marble (2). Lava stone processing was present in a few cases: three (9%) CW and only two (6%) QW. These data indicate a poor relationship between exposure to volcanic dust and pneumoconiosis.

The risk of exposure to silica from artificial marble has already been reported in other studies, which report a very high RCS content: 85–93% [5].

The analysis of the sample by work subcategories did not show any statistically significant difference.

The only difference is observed in the CW in which seven (39%) cases have an ILO classification of pneumoconiosis of type 2/2, not present in the SW and QW. In fact, in the latter, there are subcategories > 2/3. This indicates a worse pathological picture in the SW and QW compared to that of the CW.

This can be explained by the operating methods and therefore by the exposure to inhalable dust. In fact, construction work has multiple exposures that vary over time, depending on the type of interventions/works to be carried out, compared to the other two categories of workers (LSW and QW), who constantly work (40 h/week) with stone [10,26].

Respiratory symptoms were present in all subjects affected by pneumoconiosis, as well as a reduction in respiratory parameters observed in all three categories of workers, without any statistically significant difference.

From the comparison between respiratory function values and pneumoconiosis profusion (ILO category), an inversely proportional correlation is observed, in which, as the category increases, a percentage reduction in respiratory volumes is observed. Similar results were observed by Tjoe-nij et al. [10], who analyzed 1,335 workers in the construction sector exposed to RCS. Similar results were also reported in a recent study conducted by Karatas et al. [3], in which 222 cases of patients affected by silicosis were analyzed.

From the analysis of 1222 environmental samplings from RCS, Gbondo et al. [8] confirmed that the highest exposure occurs in the QW, followed by SW and CW.

As found in a recent systematic review and meta-analysis, RCLD workers affected by pneumoconiosis presented characteristics common to the majority of these patients, namely: male gender; smoker; age at diagnosis between 50–60 years; belonging to a work category that exposes to the risk of RCS; and duration of exposure > 10 years [9].

In order to analyze the effects of basaltic dust released from the processing of/with volcanic rocks from Mount Etna, 200 workers chronically exposed to such dust were analyzed.

In fact, in the eastern part of Sicily, the utilization of natural rocks, particularly lava stone sourced from quarries around Mount Etna, is widespread in the construction of houses and infrastructure.

In particular, three different categories of workers involved in the lava stone processing were analyzed: from the lava stone quarry (QW, n = 34), to professional cutters (LSW, n = 56), to workers who lay the lava stone (CW, n = 110).

The analysis of the three categories of workers, conducted in the period 2012–2022, did not reveal any statistically significant difference. For all the workers, a high percentage of smokers was evident, always >79%. Furthermore, we also noted the low use of PPE for the respiratory tract, on average 5.2 ± 4.3 years, compared to an average of years of exposure to dust of 14.2 ± 4.7.

Spirometric tests, chest X-rays, and CT scans did not show any respiratory system impairment from dust. Rather, three cases showed the typical signs of post-COVID-19 pneumonia, classic ground glass [27].

From the analysis of environmental monitoring data, a limited (low) risk of exposure to dust and in particular to respirable RCS was observed. In fact, monitoring data indicated an exposure that always remains within the limits established by law.

The RCS and CRCSE values detected in the three categories of workers exposed to volcanic dust were always low; these values are lower than those detected by Raanan et al. [5] in workers exposed to silica and affected by silicosis.

In the case of LSW, a greater exposure to total dust is detected, compared to the other two categories of workers (CW and QW). These data derive from the particular type of stone processing, which, although using cutting machines equipped with dust abatement systems (usually water), involves more detailed processing, often of a decorative nature, of the stone, carried out by workers with mechanical chisels, rotating wheels, etc. In such cases, greater dust production and therefore operator exposure may occur, due to the lack/poor abatement of dust.

Some studies have hypothesized the toxic action of volcanic dust alone or mixed with other substances/materials used for construction or with airborne industrial pollutants [28].

However, from the results obtained from this study, there does not appear to be a significant risk of exposure to RCS. This is in line with geological data indicating a concentration of SiO_2_ in basaltic rocks of approximately 50% of the weight compared to other types of rocks [29,30].

Therefore, the basaltic rocks of Etna, when worked, should generally only be a little dangerous for the health of humans and workers in particular.

Different is the case of the basaltic rocks coming from the Biancavilla area, which are contaminated by a natural asbestos-like fiber called fluoro-edenite and which have generated a case of naturally occurring asbestos (NOA) in the population living/coming from that area [31,32]. This phenomenon has led to the consequent increase in the incidence and prevalence of pathologies, including neoplastic ones, of the respiratory system both in workers and in the general population [32,33].

The most common form of silicosis is chronic silicosis, which occurs after exposure to RCS for over 10 years [8]. The exposure to silica also has the potential to impact other known health conditions like lung cancer, airway obstruction, and renal disease [5,34,35]. It must also be noted that RCS is classified as a Group 1 carcinogen by the International Agency for Research on Cancer in 1997 [8].

The use of volcanic material for building houses is a phenomenon typically present at a global level, where there are volcanoes. Therefore, it is important to consider the potential health risks associated with the extraction, processing, and use of these rocks [24]. The release of dust containing RCS during mining, cutting, and processing operations may increase the risk of occupational exposure and the development of lung diseases [24].

From the consultation of the RCLD database, there does not appear to be any correlation between pulmonary fibrosis and exposure to dust of basaltic origin. These data seem to be confirmed by the data of the health monitoring conducted over 10 years on 200 workers involved in different processes of working with lava stone.

This study has several limitations: the RCLD database may not be a reliable source from which to extrapolate data on pneumoconiosis; the number of selected cases is too low to be able to extrapolate definitive data; and environmental monitoring was not carried out every year, since in the absence of changes in work activity and therefore in the risk of exposure, in the event of values below the limits, the law does not provide for the execution of further assessments. Other limits are due to the different chemical composition of the lavas which could therefore vary the degree of exposure of workers over time. Furthermore, the number of workers monitored, especially in the LSW and QW categories, is also low. Although the diagnosis was made according to ILO classification, we recognize that X-ray alone may have limited sensitivity. Finally, the use of personal protective equipment (PPE) was reported in all work environments, but it must be emphasized that the availability of PPE does not necessarily translate into consistent or correct usage throughout the workday. Factors such as worker compliance, environmental discomfort, perceived risk, and insufficient training can significantly affect actual protection levels.

The strengths of this study are having conducted a retrospective survey on over 10,000 patients and having directly studied workers who have daily exposure to volcanic dust.

## 5. Conclusions

This retrospective study evaluated both clinical data from a reference center and surveillance findings on workers chronically exposed to basaltic volcanic dust from Mount Etna. No significant association was found between occupational exposure to this type of dust and the development of pneumoconiosis. Respiratory function tests and imaging results in the monitored workers remained within normal ranges, and environmental monitoring data consistently showed exposure levels below legal thresholds.

These findings suggest that, under current working conditions and safety standards, exposure to volcanic dust from Etna does not appear to constitute a major occupational risk for the development of silicosis or other pneumoconiosis-related conditions.

Furthermore, information/training on the obligation to use PPE for the airways, still little used today, is of strategic importance, as they are the main tool made available to the workers to protect themselves from risks present in the work environment.

Further research is warranted to monitor long-term health outcomes, especially considering possible variations in rock composition and work practices.

## Figures and Tables

**Table 1:** Main characteristics of patients of RCLD grouped by work category.

	CW	SW	QW	*p*-Value
Subjects 33 (100%)	18 (55%)	9 (27%)	6 (18%)	-
Mean age yrs	65.4 ± 4.9	66.8 ± 3.6	67.1 ± 6.7	n.s
Sex	Male (100%)	Male (100%)	Male (100%)	n.s
BMI	25.8 ± 3.2	26.1 ± 3.5	26.2 ± 2.8	n.s
Smokers	17 (%)	8 (%)	5 (%)	n.s
Pack-years	29.15 ± 11.38	28.41 ± 13.11	29.07 ± 16.92	n.s.
Age at first exposure (yrs)	18.15 ± 4.12	19.54 ± 7.37	19.19 ± 6.90	n.s.
RCS source material (n.)	Bricks (18), sandstone (18), clay (10), marble (18), cement (18), artificial stone (14), lava stone (3), and sand (15)	Marble (9), limestone rock (9), and granite (9)	Sandstone (2), marble (2), and lava stone (2)	-
Duration of dust exposure (yrs)	27.4 ± 6.8	26.7 ± 5.3	25.5 ± 3.1	n.s.
Use of airways PPE (yrs)	4.7 ± 1.2	5.3 ± 2.1	5.1 ± 1.3	n.s.
Age at pneumoconiosis diagnosis (yrs)	50.3 ± 9.2	52.1 ± 4.3	51.7 ± 8.4	n.s.
Latency of pneumoconiosis (yrs)	25.6 ± 4.8	23.3 ± 8.6	24.2 ± 6.5	n.s.
Symptoms	ChCh −/+ sputum (43/57%), dyspnea (77%), ChTi (31%), and wheezing (34%)	ChCh −/+ sputum (41/49%), dyspnea (75%), ChTi (25%), and wheezing (37%)	ChCh −/+ sputum (45/50%), dyspnea (82%), ChTi (27%), and wheezing (32%)	n.s.
Pneumoconiosis profusion category	≥2/2 (7) *; ≥2/3 (4); ≥3/2 (3); ≥3/2 (2)	≥2/3 (3); ≥3/2 (3); ≥3/2 (3)	≥2/3 (3); ≥3/2 (2); ≥3/2 (1)	*p* < 0.05 n.s.

CW = construction worker; LSW = lava stone worker; QW = quarry workers; n.c. = not calculable; n.s. = not significant; ChCh = chronic cough; and ChTi = chest tightness. * *p* < 0.05, statistically significant.

**Table 2:** Main features of workers occupational exposed to volcanic dust.

	CW	LSW	QW	*p*-Values
Workers, n (%)	110 (100%)	56 (100%)	34 (100%)	-
Age (years)	40.3 ± 6.7	41.2 ± 4.4	40.9 ± 5.2	n.s.
Sex (male)	100%	100%	100%	n.s.
Exposed to volcanic dust (years)	13.4 ± 3.9	14.5 ± 6.4	14.1 ± 2.2	n.s.
Smoking habits	90 (81%)	44 (79%)	28 (82%)	n.s.
Pack/years	28.25 ± 13.59	27.92 ± 12.21	29.01 ± 17.53	n.s.
BMI	26.9 ± 2.4	27.2 ± 2.3	26.1 ± 3.2	n.s.
Used respiratory PPE (years)	5.1 ± 3.2	5.5 ± 2.9	5.2 ± 3.4	n.s.

CW = construction worker; LSW = lava stone worker; QW = quarry workers; and n.s. = not significant.

**Table 3:** Results of instrumental investigations in workers exposed to volcanic dust.

	CW	LSW	QW	*p*-Values
FVC, %	98.2 ± 5.7	97.7 ± 1.3	96.9 ± 1.4	n.s.
FEV_1_, %	98.8 ± 7.8	96.5 ± 1.7	93.4 ± 2.3	n.s.
PEF, %	97.6 ± 7.9	96.7 ± 5.4	92.3 ± 4.6	n.s.
MEF25–75, %	94.5 ± 8.6	93.3 ± 5.2	91.5 ± 6.8	n.s.
TLC, %	96.6 ± 5.6	94.2 ± 2.9	92.8 ± 3.9	n.s.
TC findings	(2) Outcomes of COVID-19 pneumonia; (8) mild thickening of BAN hilar area	(1) Outcomes of COVID-19 pneumonia; (1) outcomes of previous pleurisy	(2) Mild thickening of BAN hilar area; (1) outcomes of previous pleurisy	n.s.
DLCO %	n.p.	n.p.	n.p.	-

CW = construction worker; LSW = lava stone worker; QW = quarry workers; n.p. = not performed (level II); n.s. = not significant; and BAN = bronchoalveolar network.

## Data Availability

The original contributions presented in this study are included in the article/Supplementary Material. Further inquiries can be directed to the corresponding author.

## Supplementary Materials

The following supporting information can be downloaded at https://www.mdpi.com/article/10.3390/jcm14113781/s1: The questionnaire administered to each subject to investigate the risk factors present in the work environment.
