# Supplementary material for: Risk of Pneumoconiosis in Workers Exposed to Crystalline Silica from Lava Rock Dust from Mount Etna
Source: J Clin Med. 2025 May 28;14(11):3781. doi: 10.3390/jcm14113781 (PMC12155804; doi:10.3390/jcm14113781)
Supplement: Supplementary file 1 [file jcm-14-03781-s001.zip › jcm-3431552-supplementary.pdf]

Structured Occupational Risk Questionnaire

(Supplementary Material - Manuscript ID: jcm-3431552)

This questionnaire complies with the principles outlined in the Declaration of Helsinki. Participation was voluntary and anonymized. The data collection was approved by the competent Ethics Committee.

Section 1: Demographics and General Information (8 items)

1. Age: ___ years

2. Gender:

- [ ] Male
- [ ] Female
- [ ] Other

3. Place of residence: ____________________

4. Current occupation: ____________________

5. Duration of employment in the current job: ___ years

6. Highest level of education:

- [ ] Primary
- [ ] Secondary
- [ ] High school
- [ ] University
- [ ] Postgraduate

7. Smoking status:

- [ ] Never smoker
- [ ] Former smoker
- [ ] Current smoker

8. Do you suffer from any chronic respiratory disease?

- [ ] Yes
- [ ] No

If yes, please specify: ____________________

Section 2: Work Environment and Exposure History (20 items)

9. Have you ever worked in an environment with airborne dust?

10. Number of years exposed to dust in your occupation: ___

11. Type of dust predominantly present (if known):

   - [ ] Silica

   - [ ] Volcanic ash

   - [ ] Coal

   - [ ] Other: __________

12. Was the dust visible to the naked eye during work?

13. Did you work indoors or outdoors?

14. Average number of hours per day exposed to dust:

15. Were dust-generating tasks part of your daily duties?

16. Frequency of those tasks:

   - [ ] Rarely

   - [ ] Sometimes

   - [ ] Often

   - [ ] Always

17. Was personal protective equipment (PPE) provided?

18. What type of PPE was used? (Multiple choices allowed)

   - [ ] Surgical mask

   - [ ] FFP2/FFP3 mask

   - [ ] Respirator with filter

   - [ ] None

19. How often did you use PPE?

20. Was PPE usage enforced by supervisors?

21. Did you receive formal training on dust risk?

22. Were there mechanical ventilation or dust extraction systems?

23. Was environmental monitoring for dust conducted at the workplace?

24. Were any warning signs or labels present about dust risk?

25. Was wetting or containment of dust used?

26. Did you share tools with others who may have handled dusty material?

27. Was smoking allowed in the workplace?

28. Was your workplace located near a volcanic zone?

Section 3: Work Practices and Occupational Health Measures (15 items)

29. Were occupational health visits scheduled regularly?

30. Were you ever tested for lung function (spirometry) during employment?

31. Have you had chest X-rays or HRCT scans related to work?

32. Have you ever been diagnosed with pneumoconiosis or suspected dust-related disease?

33. Did you ever report respiratory symptoms to occupational physicians?

34. Did you experience coughing during or after work?

35. Did you experience breathlessness during or after work?

36. Did you experience eye irritation at work?

37. Did you experience nasal irritation or frequent rhinitis?

38. Were symptoms seasonal or persistent?

39. Did you ever change job tasks due to respiratory symptoms?

40. Were other colleagues affected by similar symptoms?

41. Was there an occupational safety representative at your workplace?

42. Were workplace risk assessments shared with you?

43. Did your employer offer training on risk prevention?

Section 4: Personal Perception and Safety Culture (15 items)

44. How would you rate dust exposure in your workplace?

- [ ] Minimal
- [ ] Moderate
- [ ] Severe

45. Do you feel sufficiently informed about occupational risks?

46. Do you feel protected in your current working environment?

47. How would you rate PPE comfort and usability?

48. How would you rate the quality of safety training received?

49. Do you trust your employer's commitment to worker health?

50. How often are health and safety inspections carried out?

51. Do you know whom to contact in case of occupational health issues?

52. Have you received written communication regarding your risk exposure?

53. Do you participate in decisions regarding health and safety at work?

54. Have you ever refused a task due to health concerns?

55. Do you believe your health has been affected by workplace exposures?

56. Would you be willing to participate in further medical screenings?

57. Do you consider pneumoconiosis a relevant risk in your field?

58. Any additional comments or observations (optional): ____
